# Effects of age and extraction solvent on phytochemical content and antioxidant activity of fresh *Moringa oleifera* L. leaves

**DOI:** 10.1002/fsn3.783

**Published:** 2018-09-14

**Authors:** Pierre Nobossé, Edith N. Fombang, Carl M. F. Mbofung

**Affiliations:** ^1^ Food Biophysics, Biochemistry and Nutrition Laboratory Department of Food Science and Nutrition National School of Agro‐Industrial Sciences (ENSAI) University of Ngaoundere Ngaoundere Cameroon

**Keywords:** age of leaves, antioxidant activity, extraction solvent, *Moringa oleifera* leaves, phytochemicals

## Abstract

Antioxidant activity (AOA) and phytochemical content of *Moringa oleifera* Lam leaves were determined as a function of their age and extraction solvent. Fresh Moringa leaves aged 30, 45, and 60 days were harvested and extracted with three solvents; methanol, ethanol, and water. AOA of leaf extracts was measured using radical scavenging assays (DPPH, ABTS, antiperoxide activity [APA]) and reducing assays (FRAP and total antioxidant capacity [TAC]), and these were correlated with total polyphenols (TPC), total flavonoids (TFC), and chlorophyll contents of leaves. Significant variability (*p* < 0.05) in TPC and AOA of Moringa leaf extracts was observed with age and extraction solvent as well as their interaction. TPC and TFC increased with maturity, except in aqueous extract. The 60‐day‐old leaves showed highest TPC, TFC, and tocopherol contents with highest DPPH activity. On their part, 30‐day‐old leaves recorded better vitamin C, chlorophyll, and carotenoids with highest ABTS activity and APA. Methanol was best extraction solvent for TPC (4.6 g GAE/100 g DM) while ethanol was for flavonoids (1.8 g CE/100 g DM). Ethanol extracts exhibited the highest DPPH activity (53.3%–71.1%), while both ethanolic and methanolic extracts had similar and higher ABTS ^+^ activity (3.83–3.86 g AAE/100 g DM). Strong positive correlations (*r* ≥ 0.8; *p* < 0.05) were observed between chlorophyll content and DPPH, ABTS, and APA, suggesting that chlorophyll was the major contributor to AOA. TAC was highest in aqueous solvent. Free radical scavenging activity in Moringa leaves is positively correlated to chlorophyll, TFC, and TPC while reducing power is positively correlated to chlorophyll and TPC. AOA of fresh Moringa leaf extract is a function of its phytochemical content and is influenced by both the age of the leaves and the extraction solvent used. Methanolic and ethanolic extracts of 45‐day‐old Moringa leaves exhibited best antioxidant potentials.

## 1 INTRODUCTION

Oxidative stress is a physiological condition resulting from an imbalance between reactive oxygen species (ROS) and antioxidants in favor of the former. Oxidative stress is the underlying condition responsible for several chronic diseases such as diabetes, hypertension, inflammation, and cancer (Nimse & Pal, 2015). Management of oxidative stress involves the use of antioxidants, which are molecules intended to quench or trap ROS and prevent oxidative damage (Nimse & Pal, 2015; Sreelatha & Padma, 2009). With increasing desire to adopt healthy lifestyles, consumers are declining more and more from the use of synthetic antioxidants due to their side effects (Mar et al., 2011) in favor of dietary sources of antioxidants (Ibrahim, Mat, Lim, & Ahmad, 2013). This trend is further justified by the fact that antioxidant components from dietary sources are relatively safe.

*Moringa oleifera* Lam. (drumstick tree, horseradish tree) is an indigenous tree from Northwestern India. It has spread to Africa and is widely cultivated in Cameroon (Agamou, Fombang, & Mbofung, 2015). Moringa, which is valued mainly for its leaves, tender pods, seeds, and flowers, is considered a significant source of β‐carotene, vitamin C, minerals, and phytochemicals that exhibit a demonstrated antioxidant activity (Leelavinothan, Magdalena, Agnieszka, Anna, & Ryszard, 2007; Nobosse, Fombang, & Mbofung, 2017; Sreelatha & Padma, 2009). In addition, *M. oleifera* leaf extracts exhibit significant pharmacological activities against inflammation (Galuppo et al., 2014), diabetes and hyperglycemia (Azad et al., 2017; Fombang & Willy Saa, 2016; Jaiswal, Kumar Rai, Kumar, Mehta, & Watal, 2009), cancer (Boonsirichai & Jetawattana, 2014), and neurodegeneration (Hannan et al., 2014). Therefore, they constitute a potential material for nutraceutical formulation. The mechanisms involved in these therapeutic properties of Moringa leaf extracts are linked to their antioxidant activity among others. It is therefore necessary to master parameters that can affect antioxidant activity of Moringa leaf extracts as several factors have been shown to affect the antioxidant activity of plant materials. These are intrinsic factors such as age and cultivar and extrinsic factors such as harvesting season, locality, extraction solvent, and postharvest treatment (Agamou et al., 2015; Tlili et al., 2014). The stage of maturity is an important factor that influences the compositional quality and the quantity of phytochemicals in vegetables; due to the evidence during maturation, several biochemical, physiological, and structural modifications occur (Siddiqui et al., 2013). This could account for the inconsistent results recorded in literature with regard to antioxidant activity of plant materials in relation to the stage of maturity. Dian‐Nashiela, Noriham, Nooraain, and Azizah (2015) reported higher antioxidant activity in young *Cosmos caudatus* aqueous leaf extracts compared to mature and old leaves; Tlili et al. (2014) showed that antioxidant capacity varies significantly according to the ripening stage of *Rhus tripartitum* fruits. In the case of *M. oleifera,* it has been reported that the aqueous extract of mature leaves exhibits better antioxidant activity compared to young leaves (Sreelatha & Padma, 2009). It can thus be concluded that the optimal stage of maturity for antioxidant activity is plant‐specific. On the other hand, influence of extraction solvent on the phytochemical content and antioxidant activity of vegetables is widely reported (Do et al., 2014; Lou, Hsu, & Ho, 2014; Siddhuraju & Becker, 2003). From these studies, it appears that the notion of the best extracting solvent varies widely from one plant to another and depends on the targeted class of phytochemicals. In view of preparing nutraceuticals from *M. oleifera* leaves, it is therefore important to determine which extraction solvent will be best suited for what class of phytochemicals and the corresponding antioxidant capacity. In addition, given the variability in phytochemical contents and antioxidant activity with age of the plant, determining the optimal age for these properties will ensure efficient exploitation of the antioxidant potential of *M. oleifera* leaves. This work therefore had as aim to study the combined effect of age and extraction solvent (methanol, ethanol and water) on the phytochemical contents and antioxidant activity of *M. oleifera* fresh leaf extracts as well as the relationship between phytochemical content and antioxidant activity.

## 2 MATERIALS AND METHODS

Fresh *M. oleifera* leaves of different ages were harvested from our experimental garden in Ngaoundere, Adamawa Region, Cameroon. The trees were pruned, and fresh leaves were harvested at 30, 45, and 60 days after pruning. The harvested leaves were immediately transported to the laboratory, sorted to remove extraneous material, washed with tap water, and drained. The leaves were subsequently used for determination of antioxidant nutrients (carotenoids, tocopherols, and vitamin C), phytochemicals, and antioxidant activity.

### 2.1 Reagents

ABTS (2,2′‐azinobis(3‐ethylbenzothiazoline‐6‐sulfonic acid) diammonium salt), DPPH (2,2‐diphenyl‐2‐picrylhydrazyl), ascorbic acid, catechin, gallic acid, butylated hydroxyanisole, butylated hydroxytoluene, and potassium persulfate (dipotassium peroxodisulfate) were obtained from Sigma‐Aldrich, St Louis, USA.

### 2.2 Variation in phytochemical contents and antioxidant activity of *Moringa oleifera* leaves with age and extraction solvent

To study the effect of extraction solvent and age on phenolic content and antioxidant activity, fresh leaf samples were pounded in a porcelain mortar to a smooth paste; then 1 g of fresh sample was mixed with 20 ml of solvent (water, ethanol or methanol) in a conical flask. The conical flask was sealed with parafilm, and the mixture was stirred for 2 hr using a magnetic stirrer at room temperature (25 ± 2°C). The extracts were then centrifuged at 2500g for 30 min at 4°C (Anke DL‐6000 B; China). The pellets were re‐extracted under the same conditions with 15 ml of solvent, and both supernatants were pooled and adjusted to 40 ml with the indicated solvent. All extracts were stored at 4°C until analysis.

### 2.3 Phytochemical contents

#### 2.3.1 Total phenolic content

Total phenolic compounds in extracts were estimated as reported previously (Nobosse et al., 2017) using Folin–Ciocalteu's phenol reagent and gallic acid as standard. In brief, an aliquot (20 μl) of the extract was mixed with 0.2 ml Folin–Ciocalteu reagent (diluted in water 1:16 v/v) and 0.4 ml of 20% sodium carbonate solution. The tubes were vortexed for 15 s and allowed to stand for 40 min at 40°C for color development. Absorbance was recorded against a reagent blank at 760 nm using a UV–Vis spectrophotometer (Metertech SP8001; Germany). The total phenolic content was expressed as gallic acid equivalent (GAE) in g/100 g DM.

#### 2.3.2 Total flavonoid content

Flavonoids were determined according to the method described by Nobosse et al. (2017). Aliquots (100 μl) of Moringa extracts were mixed successively with 2.6 ml of deionized water and 0.15 ml of NaNO_2_ (5%). After incubation at 25°C for 5 min, 0.15 ml AlCl_3_ (10%) was added and the mixture was reincubated under the same conditions. At last, 1 ml of NaOH 1M was added and the absorbance was measured at 510 nm against a reagent blank. Catechin (0.01%) was used as standard, and the flavonoid content was expressed as catechin equivalent (CE) in g/100 g DM.

#### 2.3.3 Chlorophyll content

The chlorophyll content of each extract of *M. oleifera* leaves was estimated as described by Kaushal, Sharma, and Attri (2013). This consisted in the measurement of absorbance of ethanol, methanol, and aqueous extracts at 645 and 663 nm. Then, the chlorophyll content (mg/g) was calculated and further expressed in g/100 g DM as follows:

$$\text{Chlorophyll}\left(\text{mg}\;\text{per}\;\text{kg}\right)=\frac{20.2*\text{Abs}645+8.02*\text{Abs}663*V}{1,000*V}$$

where Abs645 and Abs663 are absorbance at 645 and 663 nm, respectively, V is the total volume of the extract in ml, and W is the initial mass of the sample.

### 2.4 Determination of antioxidant nutrients

#### 2.4.1 Total carotenoids and tocopherols

Carotenoids and tocopherols were extracted according to the modified AOAC, 970.64 spectrophotometric method (1974). Fresh Moringa leaves (1 g) were finely ground and extracted using 30 ml of hexane/acetone (70/30 v/v) mixture followed by filtration on filter paper (Whatman No. 1). The procedure was repeated thrice, and all extracts were pooled. The extract was poured into a separating funnel, then 50 ml of 1% sodium chloride solution was added, and the mixture was shaken and allowed to rest. The bottom acetone phase was separated from the hexane fraction. This procedure was repeated thrice, and the hexane fraction containing carotenoids and tocopherols was recovered. At last, the absorbance of this fraction was measured at 450 nm for carotenoids and 270 nm for tocopherols using a UV–Vis spectrophotometer (Metertech SP8001, Germany). The carotenoids content was calculated as β‐carotene equivalent using extinction coefficient of 2,592 (Rodriguez‐Amaya, 2001), and the tocopherols content was calculated as α‐tocopherol equivalent using extinction coefficient of 3,270 (Bell, John, Hughes, & Pham, 2014).

#### 2.4.2 Vitamin C (ascorbic acid)

Ascorbic acid was measured according to the method of AOAC (1990). The principle is based on oxidoreduction reaction between ascorbic acid and 2,6‐dichlorophenolindophenol (2,6‐DIP) in acidic medium. For extraction of ascorbic acid, 1 g of fresh Moringa leaves was triturated in a mortar in the presence of few drops of 25% acetic acid. Then, 25 ml of 25% acetic acid was added and the mixture was vigorously vortexed for 10 min. The mixture was centrifuged at 3,500 rpm for 15 min at 0°C. The supernatant was filtered through Whatman No. 1 filter paper, and the total volume was made to 30 ml with 25% acetic acid. Total ascorbic acid content in Moringa leaves was evaluated by titrimetric assay. For this purpose, 2 ml of the aforementioned leaf extract was titrated with 2,6‐DIP. The control test was realized using ascorbic acid standard 0.1% in place of the extract. The end of titration was reached when colorless ascorbic acid or extract solution turns pink in color. The results are expressed as mg ascorbic acid per 100 g DM.

#### 2.4.3 Antioxidant activity

Given that antioxidants have various mode of action such as radical scavenging and metal reduction, different assays based on these modes of action were used to determine the antioxidant activity.

#### 2.4.4 Radical scavenging ability

This was determined using DPPH, ABTS, and antiperoxide activity (APA) assays.

### 2.5 DPPH radical scavenging activity assay

The modified Brand‐Williams, Cuvelier, and Berset (1995) method was used to measure the DPPH radical scavenging activity of *M. oleifera* leaf extracts. DPPH in ethanol is a stable radical, dark violet in color. Its color is bleached by its reaction with a hydrogen donor. For analyses, 0.1 ml of each extract was added to 2 ml of 100 μM DPPH solution. The reaction mixture was incubated for 30 min in the dark at 25°C, and the absorbance was read at 517 nm, against a reagent blank. Ascorbic acid and butylated hydroxyanisole (BHA) were used as reference standards. Antioxidant activity was expressed as percentage of DPPH radical scavenged by *M. oleifera* extract and calculated as follows:

$$\text{Scavenging}\;\text{activity}\left(\%\right)=\frac{(A_{\mathrm{control}}-A_{\mathrm{extract}})*100}{A_{\mathrm{control}}}$$

where A is the absorbance at 517 nm.

### 2.6 ABTS+ radical scavenging activity

Experiments were performed according to Re et al. (1999) with slight modifications. ABTS was dissolved in water to a 7 mM concentration. ABTS radical cation (ABTS^+^) was produced by reacting ABTS stock solution with 2.45 mM potassium persulfate (final concentration) and allowing the mixture to stand in the dark at room temperature for 12–16 hr before use. For the study of Moringa leaf extract radical scavenging ability, the ABTS^+^ solution was diluted with ethanol to an absorbance of 0.700 ± 0.020 at 734 nm and equilibrated at room temperature (25 ± 2°C). A 50 μl aliquot of each extract was added to 3 ml of the diluted ABTS^+^ solution, and the absorbance reading was taken 5 min after mixing using a spectrophotometer (Metertech SP8001, Germany). Ascorbic acid was used as reference, and results are expressed as ascorbic acid equivalent antioxidant capacity (AEAC).

### 2.7 Antiperoxide activity

Antiperoxide activity was measured using the ferric thiocyanate assay as described by Miranda, Maureira, Rodríguez, and Vega‐Gálvez (2009). Two milliliters of the extract, 2 ml of linoleic acid (2.51 g/100 ml in ethanol [95%]), 4 ml of 0.05 M phosphate buffer (pH 7.0), and 2 ml of distilled water were mixed in a 10 ml screw‐top tube and maintained in a water bath at 40°C in the dark for 24 hr. A blank was prepared using 2 ml of ethanol solution containing linoleic acid, phosphate buffer (pH 7.0), and distilled water. After 24 hr of incubation, 0.1 ml of the mixture was added to 9.7 ml of 95% ethanol and 0.1 ml of 30% (w/v) ammonium thiocyanate. After 5 min, 0.1 ml of ferrous chloride (0.02 M in hydrochloric acid at 3.5 ml/100 ml) was added to the mixture and agitated. The absorbance of the mixture was measured at 500 nm using a UV–Vis spectrophotometer (Metertech SP8001, Germany). Butylated hydroxytoluene (BHT) solution was used as standard.

The antiperoxide activity of the extracts was calculated as:

$$\text{APA}=\frac{\text{Abs}\;\text{blank}-\text{Abs test}}{\text{Ab blank}}*100$$

where Abs is the absorbance at 500 nm.

### 2.8 Metal reducing ability

It was measured using the total antioxidant capacity (TAC) and FRAP assays.

#### 2.8.1 Total antioxidant capacity

The TAC was evaluated by the phosphomolybdenum assay as described by Prieto, Pineda, and Aguilar (1999). This assay is based on the reduction in Mo (VI) to Mo (V) by the sample analyzed and the subsequent formation of a green phosphate/Mo (V) complex at acidic pH. An aliquot of 0.1 ml of Moringa leaf extract was mixed with 3 ml of the reaction solution (0.6 M sulfuric acid, 28 mM sodium phosphate, and 4 mM ammonium molybdate). For the blank, 0.1 ml of the corresponding extraction solvent was mixed with 3 ml of the reaction solution. After incubation in a water bath at 90°C for 90 min, the absorbance of the test sample was measured at 695 nm against the blank. Ascorbic acid was used as standard. The antioxidant activity was expressed as ascorbic acid equivalents (AAE/100 g DM).

#### 2.8.2 Ferric reducing/antioxidant power

The ability of *M. oleifera* leaf extracts to reduce iron was determined using the FRAP method as described by Yen and Chen (1995) and modified by Nobosse et al. (2017). An aliquot of extract (1 ml) was mixed with 1 ml of 0.2 M phosphate buffer (pH 6.6) and 1 ml of 1% K_4_Fe(CN)_6_ and incubated for 20 min at 50°C. The mixture was further cooled and precipitated with 10% trichloroacetic acid solution. After centrifugation at 3,500 rpm for 15 min, 1 ml of distilled water was added to 1 ml of the supernatant and 0.1 ml of 0.1% FeCl_3_ solution was added and vortexed. The absorbance was read at 700 nm against a reagent blank. Ascorbic acid was used as reference.

### 2.9 Statistical analysis

All determinations were carried out in three replicates. Means were compared using two‐way analysis of variance (ANOVA) and separated by Duncan's multiple‐range test using Statgraphics Centurion XVI software, and *p* < 0.05 was regarded as significant. SigmaPlot 11 was used for plotting graphs. The classification and discrimination of Moringa leaf extracts as well as the correlation between phytochemicals and antioxidant activity were established by principal component analysis (PCA) using XLSTAT software (XLSTAT 2007; Addinsoft, New York).

## 3 RESULTS

This study was carried out to determine the phytochemical content and antioxidant capacity of *M. oleifera* leaves as affected by their age and the type of extraction solvent used, as well as the relationship between phytochemical content and antioxidant activity.

Phytochemical (TPC, TFC) and chlorophyll contents were significantly (*p* < 0.05) affected by the age of the leaves and the extraction solvent as well as the interactions between them (Tables 1 and 2). Total phenolic content ranged between 2.1 and 4.6 g GAE/100 g DM, and the flavonoids content ranged between 0.9 and 1.8 g CE/100 g DM (Table 1). Irrespective of age, the methanolic extract had the highest total phenolic content while ethanolic extracts had the highest flavonoids content. In methanol and ethanol, total phenolic content was lowest in 30 days and increased significantly (*p* < 0.05) in 60‐day‐old leaves, whereas in aqueous extracts, the TPC content peaked in 45‐day‐old leaves. Likewise, flavonoid content was significantly (*p* < 0.05) higher in 60‐day‐old leaves compared to 30‐day‐old leaves except in aqueous extract where no significant (*p* > 0.05) difference in flavonoid content with age was observed. In general, chlorophyll content had peaked at 30 days of maturity for all the solvents and decreased thereafter or remained unchanged (Table 1). However, aqueous extracts had significantly (*p* > 0.05) lower chlorophyll contents compared to methanol and ethanol extracts which had similar chlorophyll contents.

**Table 1:** Effect of age and extraction solvent on total phenolic, flavonoids, and chlorophyll contents of *Moringa oleifera* leaves

Extraction solvent	Total phenolic (g GAE/100 g DM)			Flavonoids (g CE/100 g DM)			Chlorophyll (mg/g)		
	30 days	45 days	60 days	30 days	45 days	60 days	30 days	45 days	60 days
Methanol	3.91 ± 0.13^l^	4.02 ± 0.21^l^	4.57 ± 0.20^m^	0.96 ± 0.05^e,f^	1.12 ± 0.14^f^	1.13 ± 0.06^f^	0.280 ± 0.025^c,d^	0.265 ± 0.001^c^	0.263 ± 0.002^c^
Ethanol	3.32 ± 0.37^j,k^	3.64 ± 0.15^k^	3.97 ± 0.17^l^	1.40 ± 0.04^g^	1.82 ± 0.08^h^	1.82 ± 0.07^h^	0.274 ± 0.002^d^	0.275 ± 0.002^d^	0.273 ± 0.00^d^
Aqueous	3.20 ± 0.10^j^	3.67 ± 0.00^k^	2.16 ± 0.31^i^	0.93 ± 0.04^e,f^	0.92 ± 0.04^e^	1.04 ± 0.07^f^	0.125 ± 0.001^b^	0.084 ± 0.008^a^	0.089 ± 0.002^a^

Note

*n* = 3, Mean ± *SD*. Values in row and column followed by different superscripts are significantly different (*p* < 0.05).

**Table 2:** ANOVA table showing effects of factors on response variables

	Source	Somme of squares	Ddl	Mean square	*F*	Probability
DPPH	A: Age of leaves	1,077.0	2	538.5	58.95	0.0000
	B: Extraction solvent	10,616.6	2	5,308.29	581.12	0.0000
	AB	202.406	4	50.6015	5.54	0.0025
	RESIDUAL	228.364	25	9.13458		
ABTS	A: Age of leaves	0.0176936	2	0.00884682	8.98	0.0020
	B: Extraction solvent	0.664608	2	0.332304	337.25	0.0000
	AB	0.0119633	4	0.00299083	3.04	0.0447
	RESIDUAL	0.0177359	18	0.00098533		
FRAP	A: Age of leaves	13.9706	2	6.98531	55.79	0.0000
	B: Extraction solvent	12.0612	2	6.03061	48.17	0.0000
	AB	8.88725	4	2.22181	17.75	0.0000
	RESIDUAL	2.25369	18	0.125205		
TAC	A: Age of leaves	0.240327	2	0.120164	7.83	0.0022
	B: Extraction solvent	5.70711	2	2.85356	185.98	0.0000
	AB	0.434112	4	0.108528	7.07	0.0005
	RESIDUAL	0.398925	26	0.0153433		
APA	A: Age of leaves	198.495	2	99.2475	9.08	0.0034
	B: Extraction solvent	1,366.53	2	683.265	62.51	0.0000
	AB	137.432	4	34.358	3.14	0.0516
	RESIDUAL	142.107	13	10.9313		
TPC	A: Age of leaves	0.623389	2	0.311694	5.61	0.0072
	B: Extraction solvent	9.02182	2	4.51091	81.15	0.0000
	AB	7.38589	4	1.84647	33.22	0.0000
	RESIDUAL	2.16779	39	0.0555844		
TFC	A: Age of leaves	0.312154	2	0.156077	22.49	0.0000
	B: Extraction solvent	4.72725	2	2.36362	340.52	0.0000
	AB	0.145145	4	0.0362862	5.23	0.0019
	RESIDUAL	0.256827	37	0.00694126		
Chlorophyll	A: Age of leaves	0.00659149	2	0.00329574	44.84	0.0000
	B: Extraction solvent	0.249585	2	0.124792	1698.00	0.0000
	AB	0.00999714	4	0.00249928	34.01	0.0000
	RESIDUAL	0.00161686	22	0.0000734938		

Notes

Probability < 0.05 is significant. TPC: total polyphenol content; TFC: total flavonoid content; CHL: chlorophyll; TAC: total antioxidant capacity; APA: antiperoxide activity; FRAP: ferric reducing/antioxidant potential.

**Figure 1:**
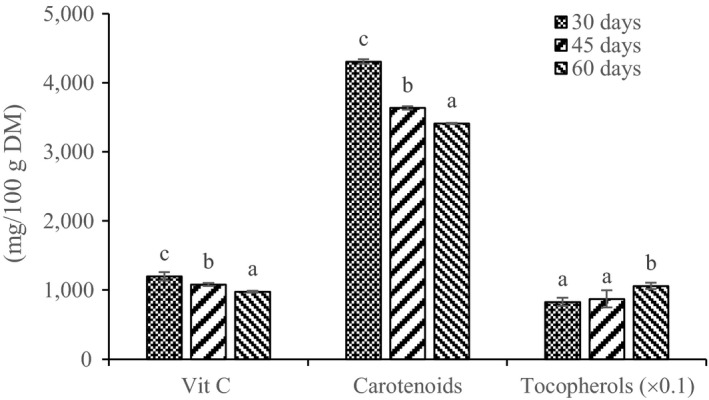
Effect of age on the vitamin C, carotenoids, and tocopherols contents of *Moringa oleifera* leaves. Different letters on the bars represent significant differences (*p* < 0.05)

Young leaves (30 days old) had the highest vitamin C content (1,178 ± 0.41 mg/100 g DM), which then decreased significantly (*p* < 0.05) with age down to the lowest value in 60‐day‐old leaves (Figure 1). Likewise, carotenoids content also decreased significantly (*p* < 0.05) as a function of leaf age from 4,300 to 3,600 and 3,400 mg/100 g DM, respectively, in the 30‐, 45‐, and 60‐day‐old leaves. On the other hand, tocopherol content increased significantly (*p* < 0.05) from 83 mg/100 g DM in 30‐day‐old to 106 mg/100 g DM in the 60‐day‐old *M. oleifera* leaves (Figure 1).

**Figure 2:**
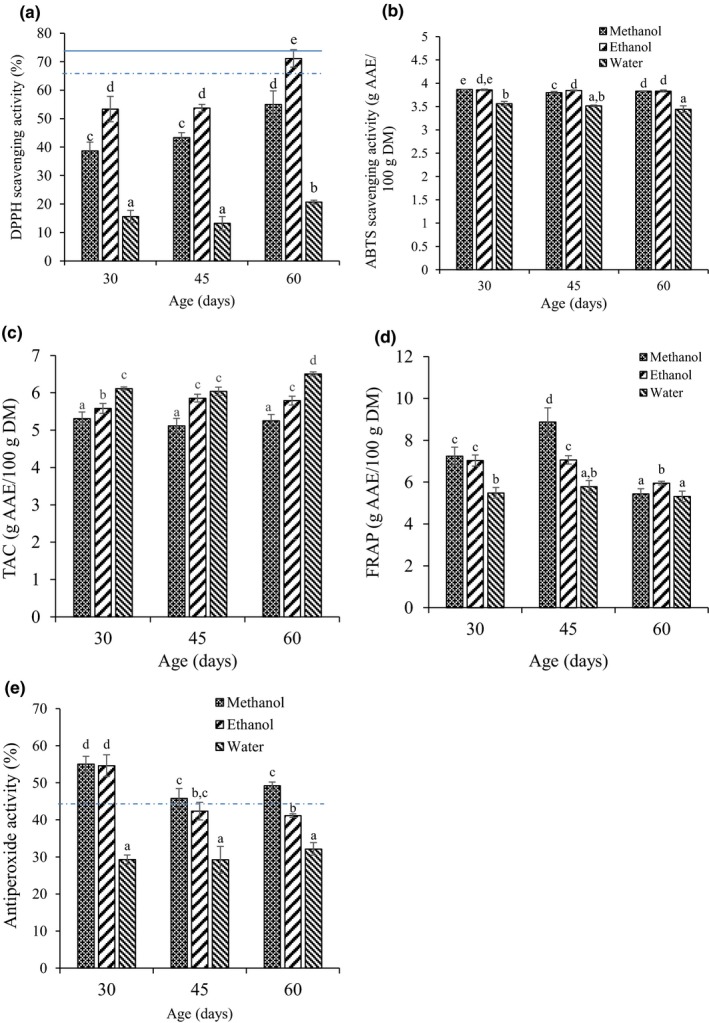
Variation in antioxidant activity of *Moringa oleifera* leaf extract with age and extraction solvent for DPPH scavenging activity (a), ABTS ^+^ radical scavenging activity (b), total antioxidant capacity (TAC) (c), ferric reducing/antioxidant power (FRAP) (d), antiperoxide activity (APA) (e). Different letters on the bars represent significant differences (*p* < 0.05). Horizontal solid and dotted line on graph represent standard vitamin C and BHT, respectively

Figure 2 presents the variation in antioxidant activity of *M. oleifera* leaves with age and extraction solvent. The multiple factors analysis of variance (ANOVA) shows that the antioxidant activity of Moringa leaf extracts is significantly (*p* < 0.05) influenced by both the age and the extraction solvent as well as their interaction except for APA where the effect of interaction was not significant (Table 2). Irrespective of extraction solvent, DPPH radical scavenging activity of *M. oleifera* leaves remained unchanged from 30 to 45 days of maturity but increased significantly (*p* < 0.05) at 60 days of maturity (Figure 2a). With respect to extraction solvent, ethanol extract had the highest DPPH scavenging activities (53.3%–71.1%) for all ages followed by methanol extracts (38.7%–55.0%) and aqueous extracts (15.6%–20.6%). The highest DPPH scavenging activity (71.1%) was observed in ethanol extract of 60‐day‐old leaves and was similar to that of 0.1 mg/ml ascorbic acid standard (73.1%) but higher than BHT standard (66.3%). In contrast to the DPPH scavenging activity, the ABTS^+^ radical scavenging activity was higher in 30‐day‐old *M. oleifera* leaves. Whereas ABTS activity of ethanol extracts did not significantly change with age, that of methanol and water extracts was significantly (*p* < 0.05) lower in 60‐day‐old compared to 30‐day‐old leaves (Figure 2b). With regard to extraction solvent, ethanolic and methanolic leaf extracts exhibited similar and significantly (*p* < 0.05) higher ABTS^+^ radical scavenging ability (3.83–3.86 g AAE/100 g DM) compared to the aqueous extract (3.44–3.56 g AAE/100 g DM).

The TAC on its part was highest in the aqueous extract of 60‐day‐old leaves (Figure 2c). Irrespective of age, the TAC varied significantly (*p* < 0.05) with extraction solvent in the order methanol (5.11–5.35 g AAE/100 g DM) < ethanol (5.58–5.85 g AAE/100 g DM) < water (6.11–6.50 g AAE/100 g DM). There were no significant age‐related differences with the methanol extracts, whereas TAC activity in both water and ethanol extracts increased with maturity. On the other hand, the ferric reducing/antioxidant power (FRAP) of Moringa leaves showed a maximum in the methanolic extract of 45‐day‐old leaves (Figure 2d). Irrespective of extraction solvent, FRAP activity was significantly (*p* < 0.05) lowest in 60‐day‐old leaves. As shown in Figure 2e, methanol and ethanol leaf extracts exhibited higher antiperoxide activity (45%–55%) at all ages compared to the aqueous extracts (27.4%–32.1%). The APA in ethanolic and methanolic extracts was highest in young leaves (30 days old) and decreased significantly (*p* < 0.05) in 45‐ and 60‐day‐old leaves; both of which had similar APA. However, the age of the leaves had no significant effect (*p* > 0.05) on APA in aqueous extracts. The butylated hydroxytoluene (BHT) solution (1 mg/ml) used as standard antioxidant exhibited an APA of 44%, which is lower than that obtained with ethanolic and methanolic extracts of 30‐day‐old leaves (55%).

**Figure 3:**
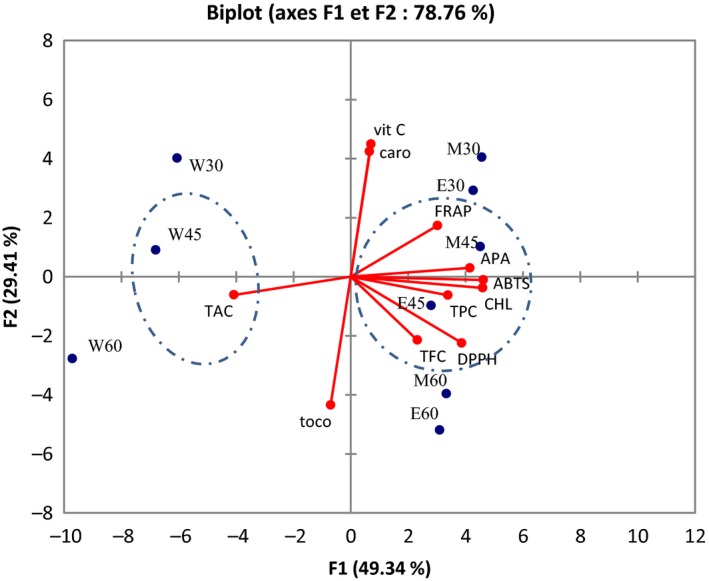
Correlation between extraction solvent, age, phytochemicals, and antioxidant activity of *Moringa oleifera* leaves. W: water extract; E: ethanol extract; M: methanol extract. 30, 45, and 60 represent the age of leaves in days. TAC: total antioxidant capacity; TP: total polyphenols; TF: total flavonoids; CHL: chlorophyll; Vit C: vitamin C; Caro: carotenoids; Toco: tocopherols; FRAP: ferric reducing/antioxidant power; APA: antiperoxide activity

The relationship between the age of the plant, extraction solvent, antioxidant activity, and bioactive compounds is represented on principal component analysis (PCA) (Figure 3). A set of eight principal components was generated by PCA. The first principal component had the highest eigenvalue of 5.4 and accounted for 49.34% of the variability in the data set. The second had eigenvalue of 3.2 and accounted for 29.41% of the variance in the data. Only principal components F1, F2 and F3 which have eigenvalues higher than 1.0 were considered significant descriptors of data variance in this study according to Kaiser's rule. Principal components F1 and F2 explain 78.76% of the observed effects. The observations were distributed and discriminated in groups having similar characteristics. The biplot correlated variables to observations. Therefore, two main groups can be distinguished (Figure 3). Group A is composed of ethanolic and methanolic extracts and is positively correlated with TPC, flavonoids, chlorophyll, DPPH, ABTS radical scavenging activity, and APA and FRAP. Groups B, characterized mainly of aqueous extracts, is opposed to Group A and shows relative positive relationship with TAC. Worldwide, the discrimination test shows that antioxidant activity in *M. oleifera* leaves is dependent on extraction solvent, age, and the nature of phytochemicals. The radical scavenging activity DPPH, ABTS, and APA is strongly correlated to TPC, TFC, and chlorophyll in both ethanol and methanol extracts of 45‐day‐old leaves. These results are supported by the Pearson correlation matrix (Table 3), which shows significant (*p* < 0.05) positive correlations between DPPH with chlorophyll (*r* = 0.88) and total flavonoids (*r* = 0.80). ABTS on the other hand shows strong significant (*p* < 0.05) positive correlation with TPC (*r* = 0.70) and chlorophyll (*r* = 0.99). APA is only significantly (*p* < 0.05) correlated with chlorophyll (*r* = 0.88) and to a lesser extent to TPC (*r* = 0.52) although not significant (*p* > 0.05). FRAP is also correlated to chlorophyll (*r* = 0.59) although not significant (*p* > 0.05). The phosphomolybdenum reducing activity (TAC) is strongly and negatively correlated with TPC (*r* = −0.82; *p* < 0.05) and chlorophyll (*r* = −0.79; *p* < 0.05).

**Table 3:** Pearson correlation matrix of phytochemicals with antioxidant activity

Variables	TPC	TFC	CHL	Vitamin C	Carotenoids	Tocopherols
TAC	−**0.820**	−0.048	−**0.791**	−0.170	−0.140	0.194
DPPH	0.547	**0.807**	**0.880**	−0.275	−0.230	0.311
FRAP	0.302	0.160	0.588	0.357	0.222	−0.518
APA	0.520	0.258	**0.881**	0.182	0.246	−0.064
ABTS	**0.698**	0.565	**0.990**	0.157	0.159	−0.134

Notes

Values in bold face are significant (*p* < 0.05). TPC: total polyphenols content; TFC: total flavonoid content; CHL: chlorophyll; TAC: total antioxidant capacity; APA: antiperoxide activity; FRAP: ferric reducing/antioxidant potential.

## 4 DISCUSSION

Antioxidant activity (AOA) and phytochemical contents of *M. oleifera* leaves showed significant (*p* < 0.05) variation with both the age of the leaves and the extraction solvent as well as their interaction (Table 2).

Methanol was the best extraction solvent for total phenolic components of *M. oleifera* leaves, whereas flavonoids were best extracted in ethanol, with 60‐day‐old leaves having the highest TPC and flavonoid contents in each case. The lowest TPC and flavonoid contents were obtained with aqueous extracts. The increase in total phenolic and flavonoid content with age can result from their active biosynthesis and accumulation in the cells during plant growth (Kong et al., 2017). This influence of age corroborates the report by Sreelatha and Padma (2009), who reported an increase in total phenolic and flavonoid contents with the age of *M. oleifera* leaves. Meanwhile, Agamou et al. (2015) observed no difference in the total phenolic content of hydroethanolic extracts of mature and young *M. oleifera* leaves. The poor extractability of *M. oleifera* leaf TPC and TFC in water compared to ethanol and methanol has been reported by several authors (Okumu et al., 2016; Siddhuraju & Becker, 2003; Vongsak et al., 2013). Contrary to our observations, Owusu‐Ansah et al. (2011) found water to be a better solvent than ethanol for the extraction of phenolics and flavonoids from Moringa leaf powder. Comparing methanol and ethanol solvents, Sultana, Anwar, and Ashraf (2009) highlighted methanol as the best extraction solvent for both phenolic and flavonoid compounds in *M. oleifera* leaves and roots. The variation of phenolic and flavonoid contents with extraction solvent could be explained by the differences in polarity and diffusion strengths of the solvents, the structural complexity, or the selective solubility of phytochemicals in a given solvent (Djeridane et al., 2006; Medini, Fellah, Ksouri, & Abdelly, 2014).

Vitamin C and carotenoids were mostly concentrated in young leaves, while tocopherols were concentrated in old leaves of *M. oleifera*. Agamou et al. (2015) reported a decrease in carotenoids content with age in Moringa leaves, which is in accordance with our observations; but in contrast observed no change in vitamin C content of *M. oleifera* leaves at two stages of maturity. Sreelatha and Padma (2009) on their part reported an increase in vitamin C, carotenoids, and tocopherol contents of *M. oleifera* leaves with maturity. The decrease observed in vitamin C and carotenoids contents with the age of the leaves may be due to metabolic degradation and utilization during plant maturation (Green & Fry, 2005), given that they are involved in cell division, plant growth, and photoprotection (Bartley & Scolnik, 1995; Smirnoff, 1996).

In addition to age and extraction solvent, the variation in AOA was also a function of the assay used. Of the five antioxidant assays tested, DPPH and TAC were maximal in extracts of 60‐day‐old leaves. Antiperoxide and ABTS activities were maximal in extracts of 30‐day‐old leaves, while ferric reducing/antioxidant power activity was highest in 45‐day‐old leaves. In general, with respect to extraction solvent, highest antioxidant activities were recorded for DPPH in ethanol; APA and FRAP in methanol; ABTS in both ethanol and methanol; and TAC in aqueous solvents.

Antioxidant activity in plant extract is generally attributed to the presence of phenolic and other antioxidant components. DPPH scavenging activity was strongly correlated to flavonoids (*r* = 0.81; *p* < 0.05) and chlorophyll (*r* = 0.88; *p* < 0.05) contents in *M. oleifera* leaves. The high DPPH activity observed with ethanolic extract of 60‐day‐old leaves may be due to their higher concentration in these components (Table 1). Sreelatha and Padma (2009) had previously reported an increase in DPPH activity with flavonoid content. ABTS activity on its part was significantly (*p* < 0.05) positively correlated to TPC (*r* = 0.70) and chlorophyll (*r* = 0.99), whereas APA was only strongly correlated to chlorophyll (*r* = 0.88; *p* < 0.05). The important contribution of chlorophyll to ABTS activity and APA may explain the high ABTS and APA observed in 30‐day‐old leaves, given that chlorophyll content peaked in these leaves. These results point to the fact that chlorophyll and flavonoids are important contributors to antioxidant activity in *M. oleifera* leaves. On the other hand, TAC was significantly (*p* < 0.05) negatively correlated with TPC (*r* = −0.82) and chlorophyll (*r* = −0.79). The high activity of TAC in aqueous medium may explain their negative association with TPC and chlorophyll as these components were least soluble in aqueous solvent. These observations are consistent with reports that antioxidant activity does not only depend on the concentration of phytochemicals but also on their composition and structural organization (Pandjaitan, Howard, Morelock, & Gil, 2005).

A better insight into the relationship between age, extraction solvent, AOA, phytochemical content, and antioxidant nutrients of *M. oleifera* leaves was obtained from PCA (Figure 3). It is worth noting that on the biplot, variables closest to one another and far from the origin are positively correlated while variables opposite of one another on the plot are negatively correlated (Shin, Craft, Pegg, Phillips, & Eitenmiller, 2010). Results showed that both ethanol and methanol extracts of 45‐day‐old leaves exhibit the most relevant radical scavenging activity (DPPH, ABTS, APA) and FRAP. Flavonoids, total polyphenols, and chlorophyll are the main components responsible for the radical scavenging activity. The DPPH scavenging activity of *M. oleifera* leaf extracts is strongly linked to the presence of chlorophyll and flavonoids as supported by the correlation between these parameters (Table 3). The ABTS radical on the other hand is preferably scavenged by both total polyphenols and chlorophyll and to a lesser extent by flavonoids in *M. oleifera* leaf extracts. Both FRAP and APA are linked to the presence of chlorophyll although FRAP is only linked to a lesser extent (Table 3). Polyphenols in general and flavonoids in particular exert antioxidant activity either by hydrogen atom transfer or by electron transfer (Di Meo et al., 2013). Both DPPH and ABTS are free radicals used to evaluate radical scavenging activity but DPPH is scavenged through hydrogen transfer from antioxidant while ABTS is scavenged through electron transfer. On the other hand, both FRAP and APA are exhibited through electron transfer from antioxidant components (Moon & Shibamoto, 2009). Moreover, DPPH is preferably scavenged by lipophilic compounds while ABTS represents the activity of both hydrophilic and lipophilic scavengers (MacDonald‐Wicks, Wood, & Garg, 2006). Thus, the hydrogen transfer capacity of flavonoids and the relatively lipophilic nature of flavonoids and chlorophyll support their high contribution to the DPPH scavenging capacity. Also, phenolic compounds are either hydrophilic or lipophilic depending on their structure, which explains their high capacity to scavenge ABTS radical.

As secondary metabolites, phenolic compounds are widely distributed in fruits and vegetables and are considered the main actors for the antioxidant capacity of plants (Tlili et al., 2014). However, the present study shows that chlorophyll is a more potent radical scavenger and reducing agent than total phenolic compounds and flavonoids. Previous studies have shown that chlorophyll exhibits free radical scavenging and metal chelating activity (Hsu, Chao, Hu, & Yang, 2013; Khattab, Goldberg, Lin, & Thiyam, 2010) and improves resistance to oxidative stress in nematodes (*Caenorhabditis elegans*) (Wang & Wink, 2016) although the mechanism of its antioxidative activity is not clear.

## 5 CONCLUSION

The phytochemical content and antioxidant activity of fresh *M. oleifera* leaves are influenced by the age of the leaves and the extraction solvent used, as well as the interaction between them. Worldwide, ethanol appears to be the most efficient solvent for the production of extracts with high flavonoid content, while methanol is better suited for production of polyphenol‐rich extracts. Both ethanolic and methanolic extracts exhibited higher antioxidant activity compared to water extracts. *M. oleifera* leaves aged 45 days are best suited for the production of extracts with the most potent antioxidant activity. The lowest antioxidant activity and phytochemical contents are obtained with aqueous extracts. AOA is a function of phytochemicals content. The free radical scavenging activity in *M. oleifera* leaves is positively correlated to chlorophyll, flavonoids, and total polyphenols while reducing power is positively correlated to chlorophyll and total polyphenols. However, chlorophyll is a more potent radical scavenger and reducing agent than total phenolic compounds and flavonoids.

## CONFLICT OF INTEREST

The authors declare no conflict of interest.

## ETHICAL STATEMENT

Human testing and animal testing were not necessary in this study.
